# The ecological suitability area of *Cirsium lineare* (Thunb.) Sch.‐Bip. under future climate change in China based on MaxEnt modeling

**DOI:** 10.1002/ece3.10848

**Published:** 2024-01-23

**Authors:** Hu‐Qiang Fang, Peng‐Fei Zhang, Shao‐Wei Xu, Teng Xu, Bing He, En Wang, Chang‐Wu Dong, Qing‐Shan Yang

**Affiliations:** ^1^ College of Pharmacy Anhui University of Chinese Medicine Hefei China; ^2^ Dexing Research and Training Center of Chinese Medical Sciences Dexing China; ^3^ The Second Affiliated Hospital of Anhui University of Traditional Chinese Medicine Hefei China

**Keywords:** *Cirsium lineare* (Thunb.) Sch.‐Bip, climate change, environment variables, habitat distribution

## Abstract

Many kinds of medicinal ingredients occur in *Cirsium lineare* that have good clinical efficacy, conferring on this species its high medicinal development value. However, with a rapidly changing global climate, it is increasingly imperative to study the factors affecting the habitat distribution and survival of species. We predicted the current and future distribution areas of suitable habitats for *C. lineare*, analyzed the importance of environmental variables in influencing habitat shifts, and described the alterations to suitable habitats of *C. lineare* in different periods (modern, 2050s, and 2070s) and scenarios (RCP2.6, RCP4.5, and RCP8.5). The results show that, under the current climate, the total suitable area of *C. lineare* is about 2,220,900 km^2^, of which the highly suitable portion amounts to ca. 292,600 km^2^. The minimum temperature of the coldest month, annual precipitation, and mean daily temperature range are the chief environmental variables affecting the distribution of habitat for *C. lineare*. In the same period, with rising greenhouse gas emission concentrations, the total suitable area will increase. In general, under future climate change, the suitable habitat for *C. lineare* will gradually migrate to the west and north, and its total suitable area will also expand. The results of this experiment can be used for the conservation and management of the wild resources of *C. lineare.* We can choose suitable growth areas to protect the medicinal resources of *C. lineare* through in situ conservation and artificial breeding.

## Graphical abstract

We predicted the distribution of *Cirsium lineare* (Thunb.) Sch.‐Bip. under future climate change by optimizing the Maxent model. The experimental results show that under future climate change, the suitable habitat for *C. lineare* will gradually migrate to the west and north, and the total suitable area will also expand.

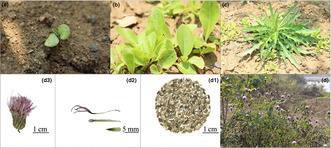

## 1 INTRODUCTION

In their fifth assessment report, the Intergovernmental Panel on Climate Change (IPCC) pointed out that, from 1951 to 2010, due to large amount of greenhouse gases released via human activities, the global average surface temperature has increased considerably, triggering a series of extreme climatic changes such as glacier melting and rising sea levels (IPCC, 2014). Global warming will continue to intensify. Environmental variables are critical factors affecting the survival and development of species, significantly shaping the spatial distributions of animal and plant habitats (Bertrand et al., 2011). Studies have shown that climate change may affect the geographical distribution of certain plants and the timing of flowering and fruiting for certain species (Correa‐Lima et al., 2019). For example, under future climate change, the suitable growth area and area of *Ephedra sinica*, *Codonopsis pilosula*, *Opuntia streptacantha*, and other plants will change significantly (Cruz‐Jimenez et al., 2023; Guo et al., 2023; Shaban et al., 2023; Wan et al., 2021; Wang et al., 2019). The environment interacts with the distribution of plant habitats, in that environmental changes will drive the migration of suitable distribution areas for plant species, and the modified geographical distribution of these plants will in turn determine the ecological environment of their habitats (Zhang et al., 2018).

In recent years, the maximum entropy model (MaxEnt model), Maxlike, Biomod2, and other modeling methods combined with geographic information spatial analysis technology have been widely used to assess the impact of climatic environmental variables on the potential distribution of species (Fetene et al., 2022; Merow & Silander, 2014; Pant et al., 2021) and further used to comprehensively analyze trends in these distributional changes under past or future climate change (Liu et al., 2021; Pan et al., 2022). MaxEnt model is commonly used for species distribution modeling, and its performance is excellent. It employs machine learning technology and an underlying calculation formula that is simple and accurate. Research has shown that even if the coordinate information of distribution points is inaccurate and the correlations between climate and environmental parameters are unpredictable, MaxEnt is still useful. Importantly, the MaxEnt model is superior to other models when the available sample size is limited (Schmidt et al., 2020; Zeng et al., 2016). For example, Chen et al. (2022) used the MaxEnt model to predict the distribution of *Pomatosace filicula* on the Qinghai–Tibet Plateau under climate change. Their findings showed that future climate change will increase the risk posed to the survival of this species, whose habitat will be reduced further and degraded (Chen et al., 2022). Using the MaxEnt model, Zhang et al. (2022) were able to identify and locate suitable habitats of *Wolfiporia cocos* distributed mainly in China's Yunnan Province. Accordingly, MaxEnt has become one of the most widely relied‐upon tools for studying the distribution of species. Its perquisite conditions for operation are relatively low, requiring only a small amount of species distribution data, along with continuous or sub‐type climate variable data as input parameters (Anand et al., 2021; Elith et al., 2011; Guo et al., 2017; Morales et al., 2017; Song et al., 2021).

**FIGURE 1:**
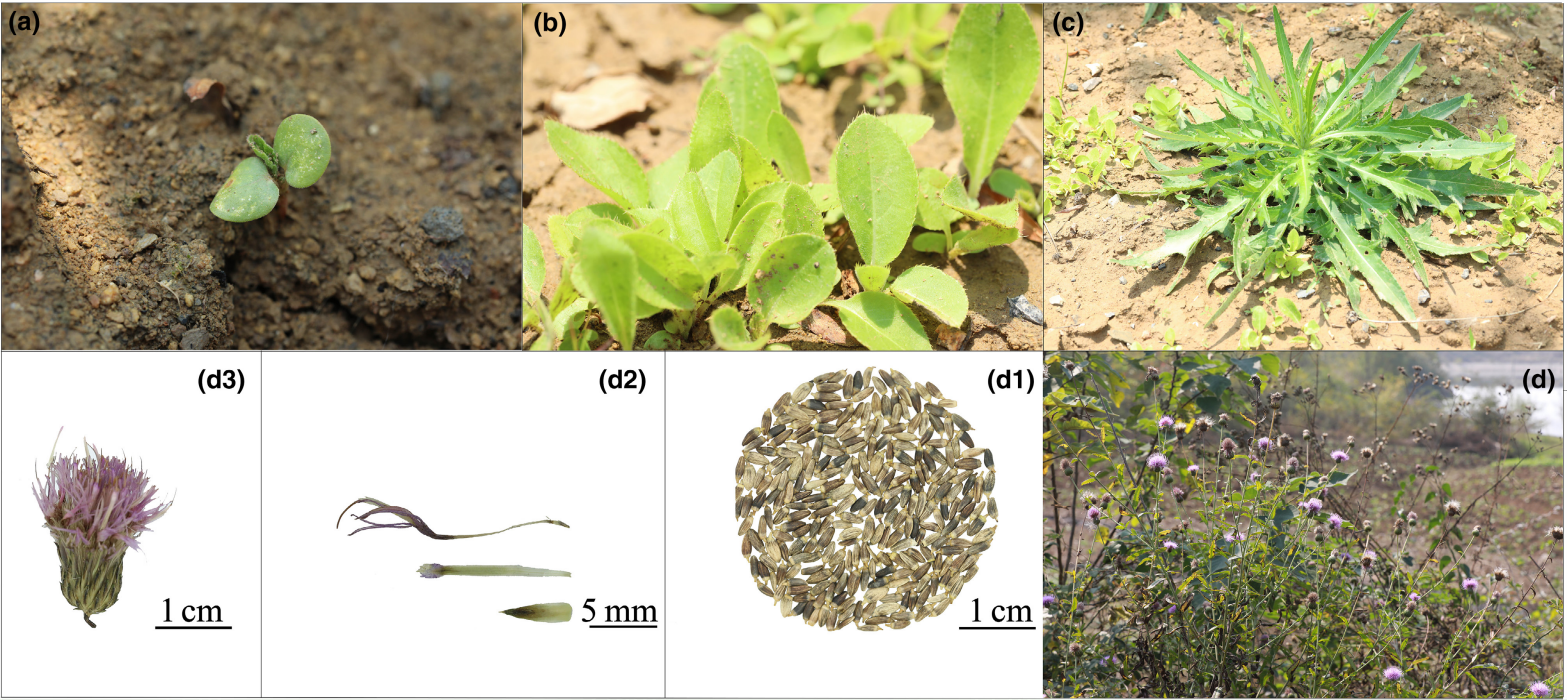
Plant morphology and reproductive organs of *Cirsium lineare*. (a) Scion; (b, c) seedling; (d) adult plants; (d1–d3) lean fruits and cephalic inflorescences.

*Cirsium lineare* is a plant in the family Asteraceae, and its leaf shape varies greatly at different growth stages (Figure 1a–c). Furthermore, its fruit is an achene (Figure 1d1), with crown hair, which can be carried away airborne by the force of wind, which is conducive to seed (achene) dispersal and propagation (seedling recruitment). Notably, the roots of *C. lineare* contain *n*‐heptadecanol, stigmasterol, β‐stigmasterol, and other chemical components (Liu et al., 2009), whose use can significantly relieve uterine contraction and has certain anti‐inflammatory effects (Li, 2000). In modern clinical practice, the plant's bioactive extracts are mainly used to treat irregular menstruation, amenorrhea, and dysmenorrhea, among other ailments (NJUCM, 1997).

With the mining of its wild populations and application of *C. lineare*'s medicinal value, its artificial planting resources are being gradually developed. We found through field investigations that the distribution and resources of wild *C. lineare* are diminishing due to factors such as mountain closure and afforestation, socio‐economic production and construction, and the use of herbicides. Presently, the *C. lineare* plantation industry remains under development, but it lacks specific and proven management practices, resulting in the serious overexploitation of *C. lineare* populations. This phenomenon of drastic resource loss has already occurred in some areas. As a perennial medicinal plant, the wild populations of *C. lineare* are not easily replenished in a short time, and the acquisition of this plant's medicinal resources is currently unsustainable (Applequist et al., 2020). Furthermore, ongoing climate change will likely not only affect the storage of medicinal plant resources, but also their chemical composition (Albert et al., 2009; Shen et al., 2021), and drive spatiotemporal changes in the natural habitat of plant species they are extracted from. Yet the natural distribution of *C. lineare* is surprisingly understudied. Investigating it in depth would be of great significance for knowing the suitable distribution of *C. lineare*, to effectively protect its plant resources, and for providing a reference basis for determining the production area of high‐quality medicinal materials (Wan et al., 2021; Wei et al., 2018).

In this study, we used the MaxEnt model to examine the relationship between the current and future geographical distributions of *C. lineare* in response to climate change. This research quantifies both current and future distribution areas of this plant, their spatial changes and trends over time, and the contribution from environmental variables. Through this study, we can (1) identify the dominant environmental variables affecting the distribution of *C. lineare*; (2) demarcate, based on current climate conditions, the distribution of suitable habitat for *C*. *lineare*; and (3) determine, against the background of future climate change, the distribution and changes to suitable habitat areas for *C. lineare*.

## 2 MATERIALS AND METHODS

### 2.1 Data collection

#### 2.1.1 Distribution data

**FIGURE 2:**
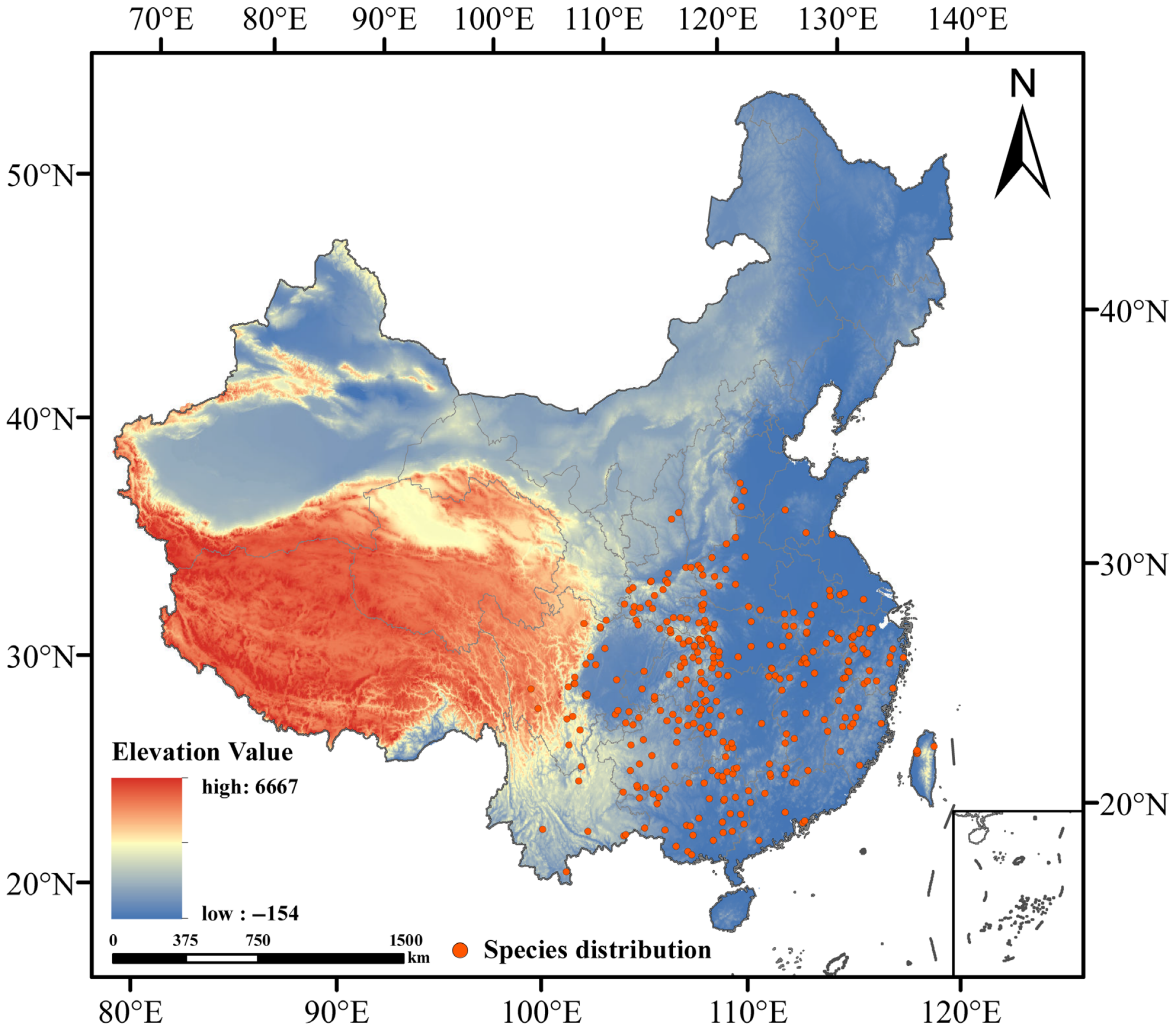
Distribution of the sample points of *Cirsium lineare* in China.

Through the Chinese Virtual Herbarium (http://www.cvh.ac.cn/; accessed on October 20, 2022), the Global Biodiversity Information Service Network Platform (https://www.gbif.org/; accessed on October 20, 2022), and the Fourth Chinese Materia Medicine Resources Survey, we collected presence data of *C. lineare* between 1960 and 2022, to describe its distribution across China. For the distribution points lacking exact longitude and latitude coordinates, we used Google Maps to query the coordinate information of their collection sites (Ji et al., 2021). Finally, in this way, we obtained a total of 605 geographical distribution data points. To avoid over‐fitting the model from too dense a distribution of points, which would affect the results' accuracy, we divided the study area into grid cells—each covering 2.5′ × 2.5′—with only one distribution point selected in each grid cell (Rodriguez‐Castaneda et al., 2012). After deleting those distribution points with repeated or similar longitude and latitude values, we were left with 315 pieces of valid information regarding the distribution points of *C. lineare*. Finally, we converted these distribution points into latitude and longitude coordinate information and mapped them as the sample points (Figure 2).

#### 2.1.2 Environmental variables’ data and sources

The climate data used in this study all came from WorldClim online (http://www.worldclim.org/; accessed on November 15, 2022) at a 2.5‐min resolution. This included 19 bioclimatic variables (v1.4) in the modern (from 1960 to 1990) scenario and future bioclimatic variable data (CCSM4) under three different scenarios in the 2050s (averaged for 2041–2060) and 2070s (averaged for 2061–2080) from the CCSM4 climatic system model, which was released by Phase 5 of the Coupled Model Intercomparison Project (CMIP5).

RCPs are a series of comprehensive greenhouse gas concentration and emission scenarios, which are often used as input parameters of climate change prediction models under the influence of human activities in the 21st century (Moss et al., 2010). They are also scenario models of greenhouse gas emissions of different concentration levels as adopted by the IPCC in their fifth assessment report (IPCC, 2014). These include RCP2.6, RCP4.5/RCP6.0, and RCP8.5 (Remya et al., 2015), which denote low‐, medium‐, and high‐concentration greenhouse gas emission scenarios, respectively. Both RCP4.5 and RCP6.0 are considered medium greenhouse gas emission scenarios, but the former has higher priority (Gebrewahid et al., 2020). In the end, we selected RCP2.6 (minimum greenhouse gas emission), RCP4.5 (medium greenhouse gas emission), and RCP8.5 (maximum greenhouse gas emission) to simulate the distribution of suitable growth habitat areas of *C. lineare* in the future (Ma & Sun, 2018).

### 2.2 Data analysis

#### 2.2.1 Environmental variable selection

**FIGURE 3:**
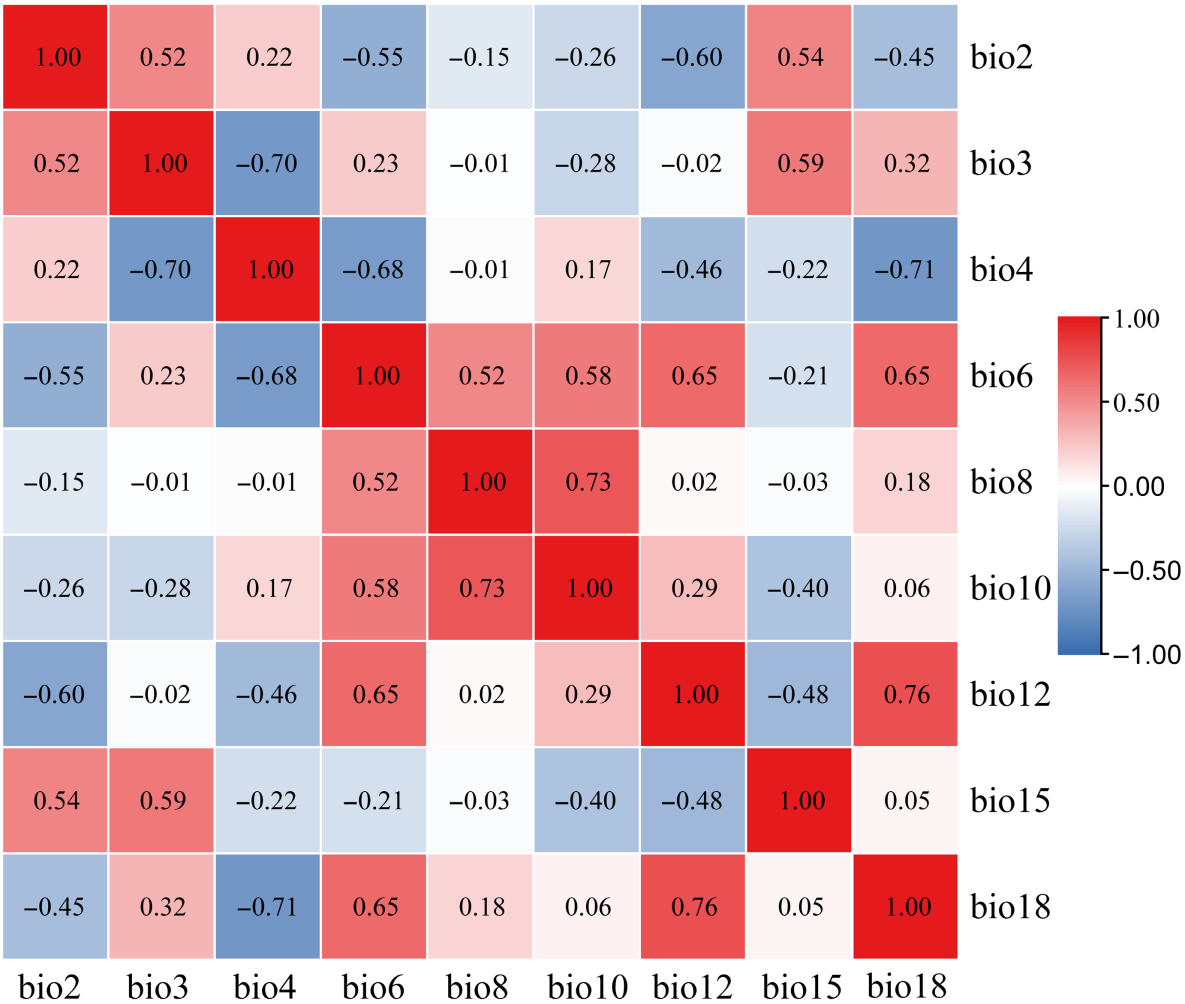
Heat map of the Pearson correlation of the nine environment variables involved in MaxEnt modeling. The explanations of these environmental variables are provided in Table 1.

Environmental variables have a pivotal impact on the analysis of predicted modeling results and the determination of the environmental niche for species suitability (Marshall et al., 2018). Among the 19 environmental variables used here, there are usually several variables strongly correlated with each other (Pascoe et al., 2019). To lessen the impact between environmental variables and reduce their error, we imported all 19 of them along with the *C. lineare* sample distribution data into the MaxEnt model software (www.cs.princeton.edu/~schapire/MaxEnt; accessed on October 20, 2022) for a pre‐experiment pilot exercise and obtained the respective contribution values of the 19 environmental variables. Meanwhile, ArcGIS v10.4 software (http://www.arcgis.com; accessed on October 20, 2022) was used to extract the 19 environmental variables' data per distribution sample point, and their pairwise Pearson's correlations were tested in IBM SPSS Statistics 20 software (https://www.ibm.com/cn‐zh; accessed on November 19, 2022). When the correlation coefficient between two environment variables was high (|*R*| ≥ 0.8) (Shcheglovitova & Anderson, 2013), we eliminated the variable having the lower contribution value. In this way, we settled on 9 of the initial 19 environmental variables for use in the model predictions; their correlation heat map is shown in Figure 3.

#### 2.2.2 Model parameter optimization and modeling

Research has shown that setting default parameters when the MaxEnt model is running may lead to its poor prediction performance (Cobos et al., 2019). The prediction performance and accuracy of this model are affected by two key parameters: the regularization multiplier (RM) and the feature combination (FC). Given that different combinations of RM and FC will generate differing models, we used the “kuenm” package in R v3.6.3 to optimize the model (Liu & Shi, 2020) and then adjusted these two parameters accordingly in MaxEnt to yield the best model.

We used 75% of the screened distribution data for mode training and reserved 25% for testing. The RM constant was set to 0.1–4.0, a step size = 0.1, and there were 40 values in total. FC was composed of five feature categories: l (linear), q (quadratic), p (product), t (threshold), and h (hinge), with a total of 29 combination types: l, qp, qt, lqpt, and so forth. Different RM constants and FC were combined into 1160 models. The kuenm package can automatically analyze each model and derive its performance parameters. We selected the best model according to the value of these parameters. The best model should have the following characteristics: (1) be statistically significant; (2) an omission rate ≤5%; and (3) an AICc value that is minimal (or ΔAICc is 0). Finally, the model that met all the above characteristics to the fullest was chosen (Wu et al., 2021).

We imported the sample distribution data (in CSV format) and the nine filtered environment variables into the MaxEnt model and set its parameters according to the above model optimization results. Next, we randomly divided the sample distribution data into 75% training vs. 25% testing and set the repetition to 10 times (Fourcade et al., 2014). Finally, resulting data obtained from the model operation were analyzed. We used the area under the curve (AUC) of the receiver operating characteristic curve to evaluate the predictive performance of the model (Li et al., 2019). AUC is widely used to evaluate the performance of a variety of models and is not affected by the threshold setting. Usually, its value is taken as an indicator of the prediction effect of the model, and its range is usually 0.5–1 (Zhao et al., 2021). In the case of 0.5 ≤ AUC < 0.6, model performance is unqualified; 0.6 ≤ AUC < 0.7 means the model performance is poor; 0.7 ≤ AUC < 0.8 means the model performance is average; 0.8 ≤ AUC < 0.9 means the model performance is good; and 0.9 ≤ AUC < 1 means the model performance is excellent (Aidoo et al., 2022; Zhang et al., 2019). The model's resulting AICc value is a reliable indicator for evaluating its complexity, while the AUC value is used to evaluate its prediction accuracy. Both can be used to gauge the prediction performance of the model.

#### 2.2.3 Classification of suitable habitats

We imported the result data generated by MaxEnt into ArcGIS v.10.4 and applied the latter's re‐classification tool to manually classify the fitness grade of the habitat prediction results of *C. lineare*. The classification ranged from 0 (least suitable) to 1 (most suitable), and P represented the suitability index of the distribution of *C. lineare*. Based on Maxent's operating results, we used the maximum test sensitivity plus specific (MTSPS = 0.3029) as a threshold for dividing suitable and unsuitable zones (Brown et al., 2017), and on this basis, the suitable areas were divided into three levels. Therefore, the classification results were as follows: P < MTSPS (unsuitable area); MTSPS ≤ P < 0.45 (low‐suitability area); 0.45 ≤ P < 0.60 (mid‐suitability area); and 0.60 ≤ P (high‐suitability area).

#### 2.2.4 Future changes in suitable habitat areas and centroids

To further study the trends in changes to suitable areas for *C*. *lineare*, the SDM toolbox v2.4 (in the ArcGIS toolbox) was used to calculate the change to the total suitable area, high‐suitable area, and their centroids under different periods and scenarios in the present and future (Yan et al., 2021). Next, ArcGIS was used to map and analyze the altered geographical distribution pattern, centroid changes, and migration distance of suitable habitat for *C. lineare*. The changes in the habitat's geographical distribution pattern reflected the changes to suitable habitat areas for the plant, and the shift in the centroid reflected the trend of suitable habitat change across space and time.

## 3 RESULTS

### 3.1 Optimal model parameters and accuracy evaluation

**FIGURE 4:**
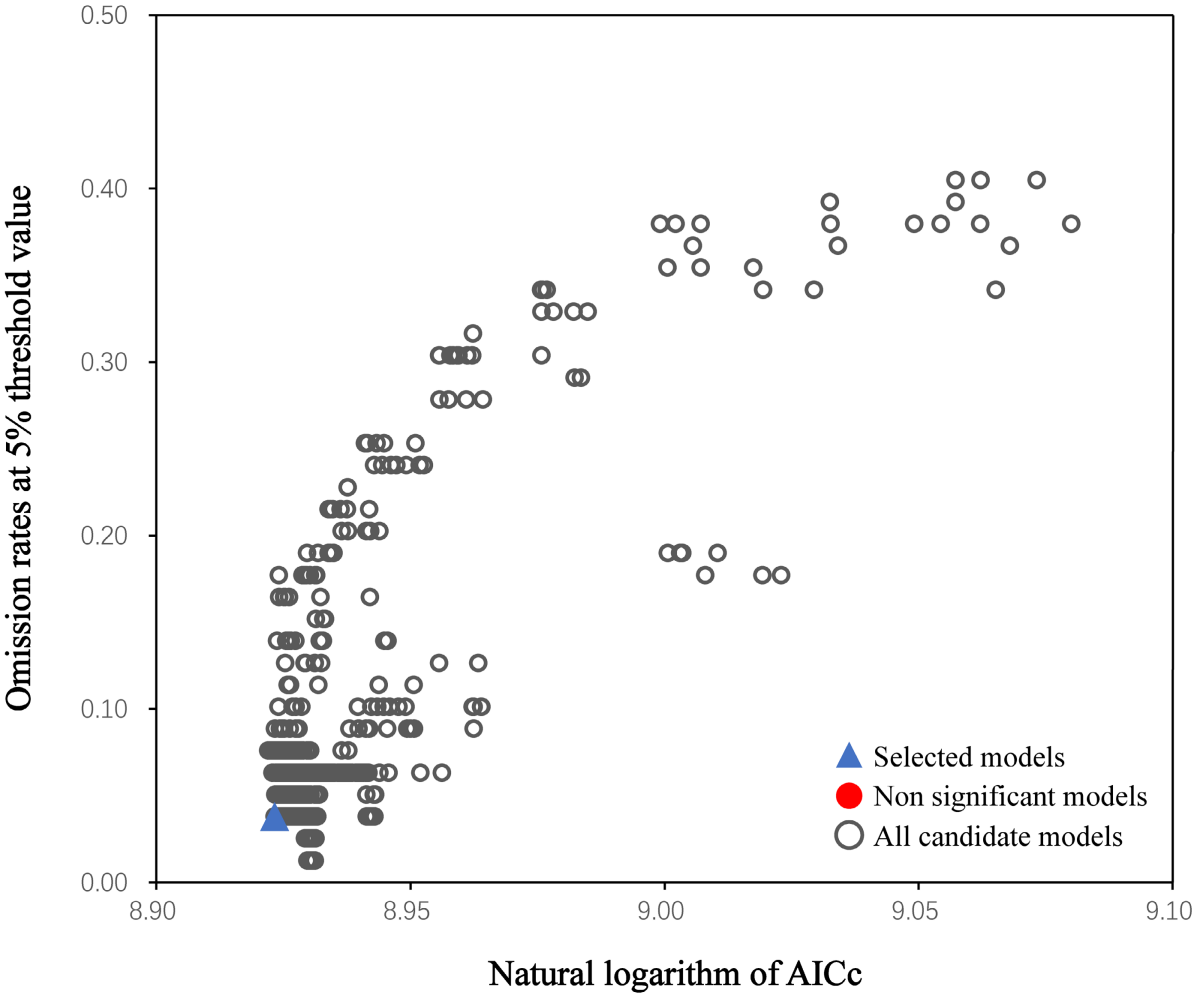
MaxEnt model parameter optimization results.

The MaxEnt model was used to predict the potential distribution area of *C. lineare* in different periods and situations in China. When the default parameters were run (FC = LQPTH; RM = 1), the ΔAICc was 45.2625 and the omission rate was 0.0886. When the model was run with optimized parameters (i.e., *F* = 1.8; RM = LQPT), the ΔAICc was 0, and the omission rate was 0.0380. This showed that the prediction accuracy of the optimized model was high and the degree of over‐fitting was low (see Figure 4 for the model optimization results). Therefore, the method of model optimization was successful and gave high accuracy for predicting the suitability distribution of *C. lineare* in China under the different climate scenarios explored here.

### 3.2 Analyzing the importance of environmental variables

**FIGURE 5:**
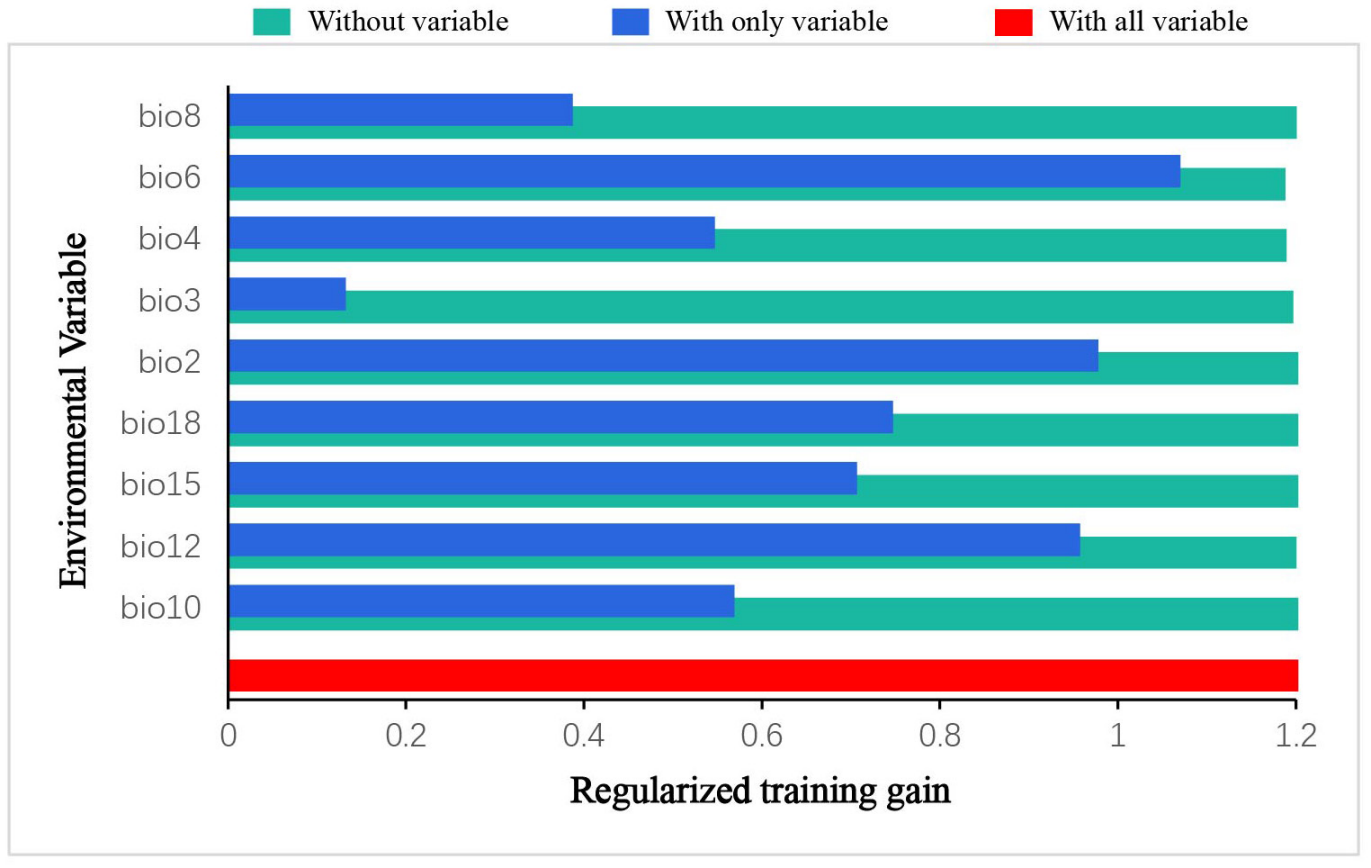
Jackknife testing of environment variables involved in the modeling (regularized training gain). The explanations of these environmental variables are in Table 1.

After the MaxEnt model had been successfully established and run, we used the jackknife method to analyze the importance of various environmental variables according to the generated result data. This revealed that bio6 (76.8%), bio12 (7.4%), and bio2 (6.4%) had the highest contribution rates among the environmental variables used to build the model, with the cumulative contribution of the three factors reaching 90.6% (Table 1). According to these jackknife test results, when using a single environmental variable to run the model, the regularized training gain value, test gain value, and AUC value of bio6 and bio2 environmental variables were generally the largest (Figure 5). Hence, bio2 (mean diurnal range), bio6 (min temperature of coldest month), and bio12 (annual precipitation) were deemed the main environmental variables that affected the suitability distribution of *C. lineare*.

**TABLE 1:** The percentage contribution and permutation importance of environmental variables of *Cirsium lineare* in the fitted MaxEnt model.

Symbol	Environmental variable	Percentage contribution (%)	Permutation importance
Bio6	Minimum temperature of coldest month	76.8	52.7
Bio12	Annual precipitation	7.4	13.2
Bio2	Mean diurnal range (mean of monthly max temp – min temp)	6.4	1.5
Bio4	Temperature seasonality (standard deviation × 100)	4.3	11.1
Bio3	Isothermality (BIO2/BIO7) (×100)	2.3	5.4
Bio15	Precipitation seasonality (coefficient of variation)	2.2	3.1
Bio8	Mean temperature of wettest quarter	0.3	7.5
Bio10	Mean temperature of warmest quarter	0.3	4.1
Bio18	Precipitation of warmest quarter	0.1	1.2

### 3.3 Current potential distribution

**FIGURE 6:**
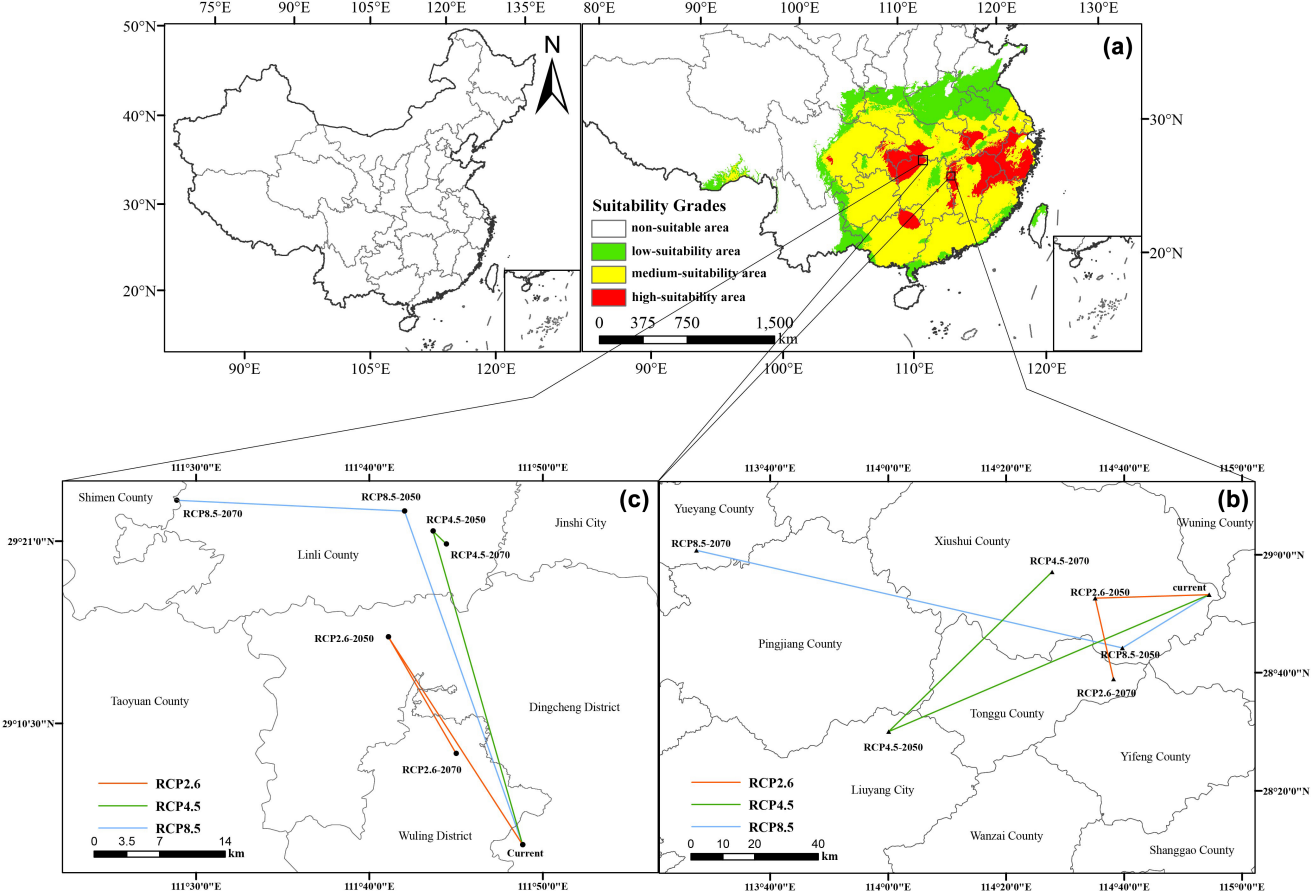
Current potential habitat distribution and centroid migration. (a) Current potential distribution area; (b, c) centroid migration under three different climate scenarios.

According to MaxEnt modeling results, combined with the above classification method of fitness grade, we drew the current potential distribution map of *C. lineare* in China (Figure 6a). The white area was not suitable for growth of *C. lineare* (0.00–MTSPS); the green area corresponded to the low‐suitability growth area (MTSPS–0.45); the yellow area indicated mid‐suitability for growth (0.45–0.60); and the red area represented the high‐suitability area (0.60–1.00). According to that map, the total suitable potential distribution area (i.e., summing low‐, medium‐, and high‐suitable areas) of *C. lineare* was about 2,220,939 km^2^, accounting for 23.1% of the country's entire land area, being mainly located in East China, Central China, the southeastern part of Northwest China, the eastern part of Southwest China, and South China.

The highly suitable area for *C. lineare* covered 2,926,304 km^2^ or 3.1% of the entire country. This habitat was mainly located in seven provinces and cities (autonomous regions), including Zhejiang Province, south‐central Anhui Province, southwestern Hubei Province, southern Chongqing City, northwestern Hunan Province, eastern and western Jiangxi Province, and northeastern Guangxi Province.

### 3.4 Suitable habitat area under global warming scenarios

In this experiment, we predicted the potential suitability distribution of *C. lineare* in China under six different scenarios. These results showed that, under future climate change, where the total suitable distribution area of *C. lineare* is found will change little overall, being mainly in East China, Central China, southeast of Northwest China, eastern part of Southwest China, and South China. Under the three climate scenarios, compared with the current state, the total coverage of suitable areas increased by 5.3% (RCP2.6), 6.7% (RCP4.5), and 9.4% (RCP8.5) during 2041–2060; afterward, during 2061–2080, it increased by 3.0% (RCP2.6), 7.1% (RCP4.5), and 11.1% (RCP8.5). In summary, the total suitable growth area of *C. lineare* will expand in the future. Notably, under the three scenarios of greenhouse gas emission concentrations through to the 2080s, the maximum attainable suitable habitat will reach 2,467,210 km^2^.

Unlike the change in the total suitable area, the relative proportion corresponding to high‐suitability area was not stable. It decreased from the current 3.0 to 2.8% and then increased to 4.1% of the entire country's area under the RCP2.6 scenario. Under the RCP4.5 scenario, the total high‐suitability area first increased to 5.4% and then decreased to 4.065%. Under the scenario of RCP8.5, its total area first increased from 3.0 to 3.5% and then decreased to 2.9%.

**FIGURE 7:**
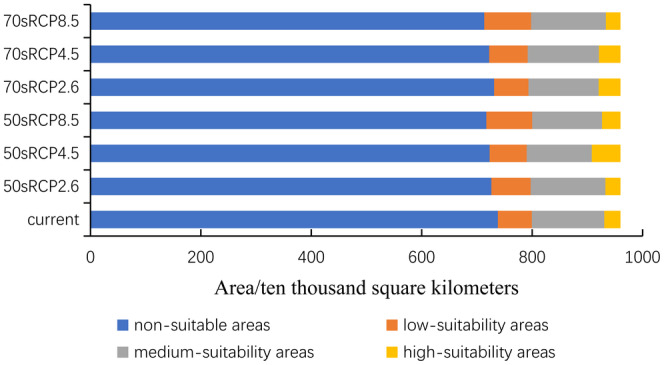
Changed area of suitable habitat under three climate scenarios (RCP2.6, RCP4.5, and RCP8.5). The three periods are current, the 2050s, and the 2070s.

Comparing now the climate scenarios for the same time period, in the 2050s, with the gradual increase of greenhouse gas concentrations, the scope of non‐suitable areas continued to shrink, whose relative proportion fell slightly from 75.642% to 75.308 and 74.7%, respectively. In the 2070s, the proportion of unsuitable areas also decreased from 76.2 to 75.2% and 74.3%, respectively. In general, within a certain range, with rising greenhouse gas concentrations, the non‐suitable area will eventually turn into suitable area, and the total suitable area of *C. lineare* in China will expand incrementally. The changes in the area of suitable and unsuitable zones in the different periods and scenarios can be seen in Figure 7.

### 3.5 Analysis of changes in the distribution pattern of habitat

**FIGURE 8:**
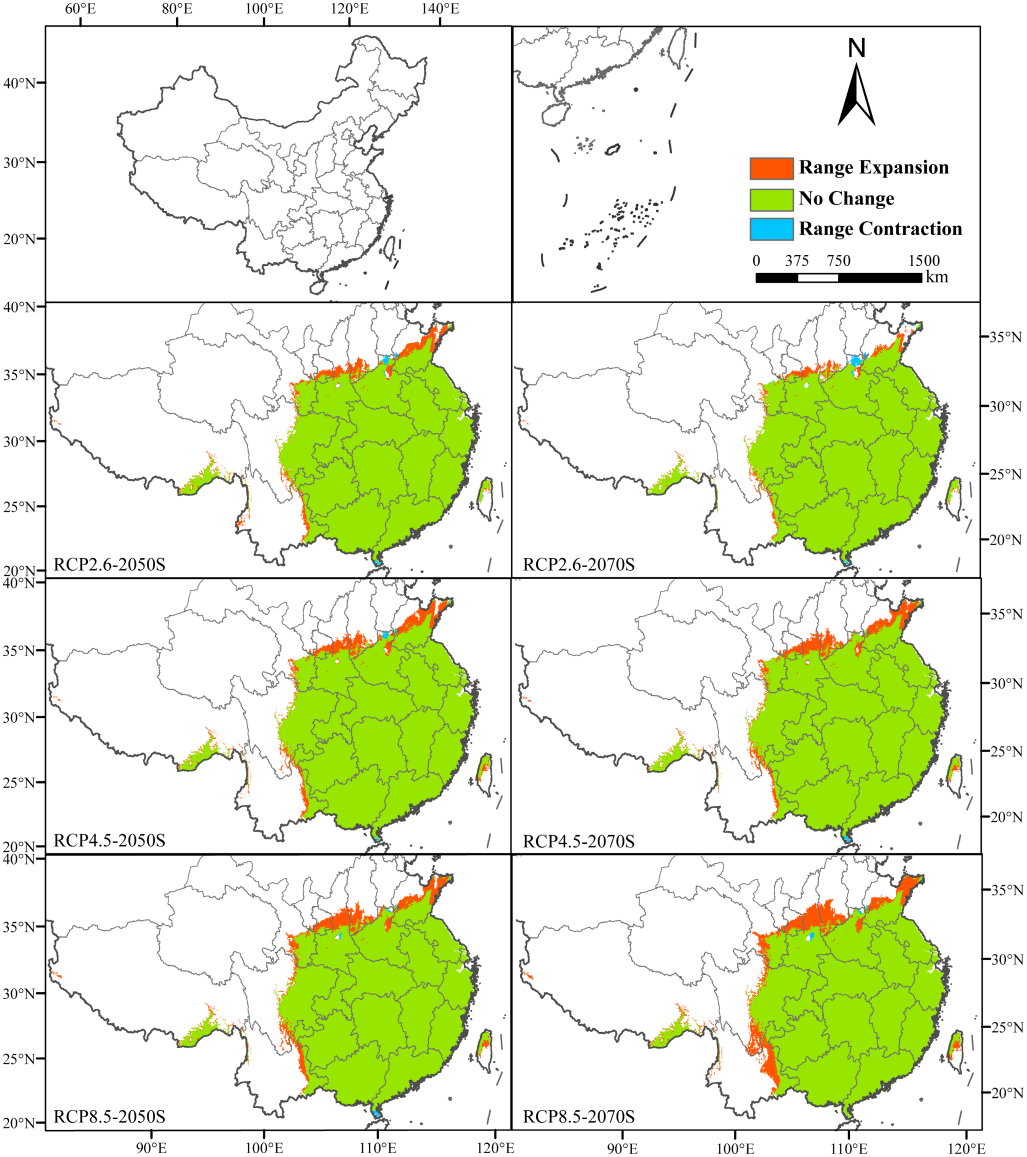
Spatial and geographical pattern change of the total suitable habitat in the 2050s and 2070s compared with the current (red represents expansion area, green represents stability area, and blue represents contraction area).

Before studying the altered spatial pattern of future suitable habitat of *C. lineare*, we installed the SDM toolbox and applied its distribution change tool to analyze the distance and direction of the change in total habitat and the high‐suitability habitat areas and generated the track map of the centroid change (Figure 6b,c). When taking the MTSPS value (=0.3029) as the threshold (Figure 6c) to study the changed total suitable growth area, all centroids are located in Changde City, Hunan Province. The current period's centroid is located in Dingcheng District, Changde City (111.8140°E, 29.0583°N). In the future, the centroid in each scenario will move northwest relative to the current centroid's position. From the current period to the 2050s, the centroid of RCP2.6, RCP4.5, and RCP8.5, respectively, moved 25.46, 34.51, and 37.28 km toward the northwest, but from different angles. From the 2050s to the 2070s, under the RCP2.6 and RCP4.5 scenarios, the centroid moved 13.94 and 1.87 km to the southeast, respectively. Under the RCP8.5 scenario, the centroid moved 21.14 km to the northwest at a small angle. In general, under the emission scenarios of various greenhouse gas concentrations, the total suitable area of *C. lineare* will expand to the west and north (Figure 8).

**FIGURE 9:**
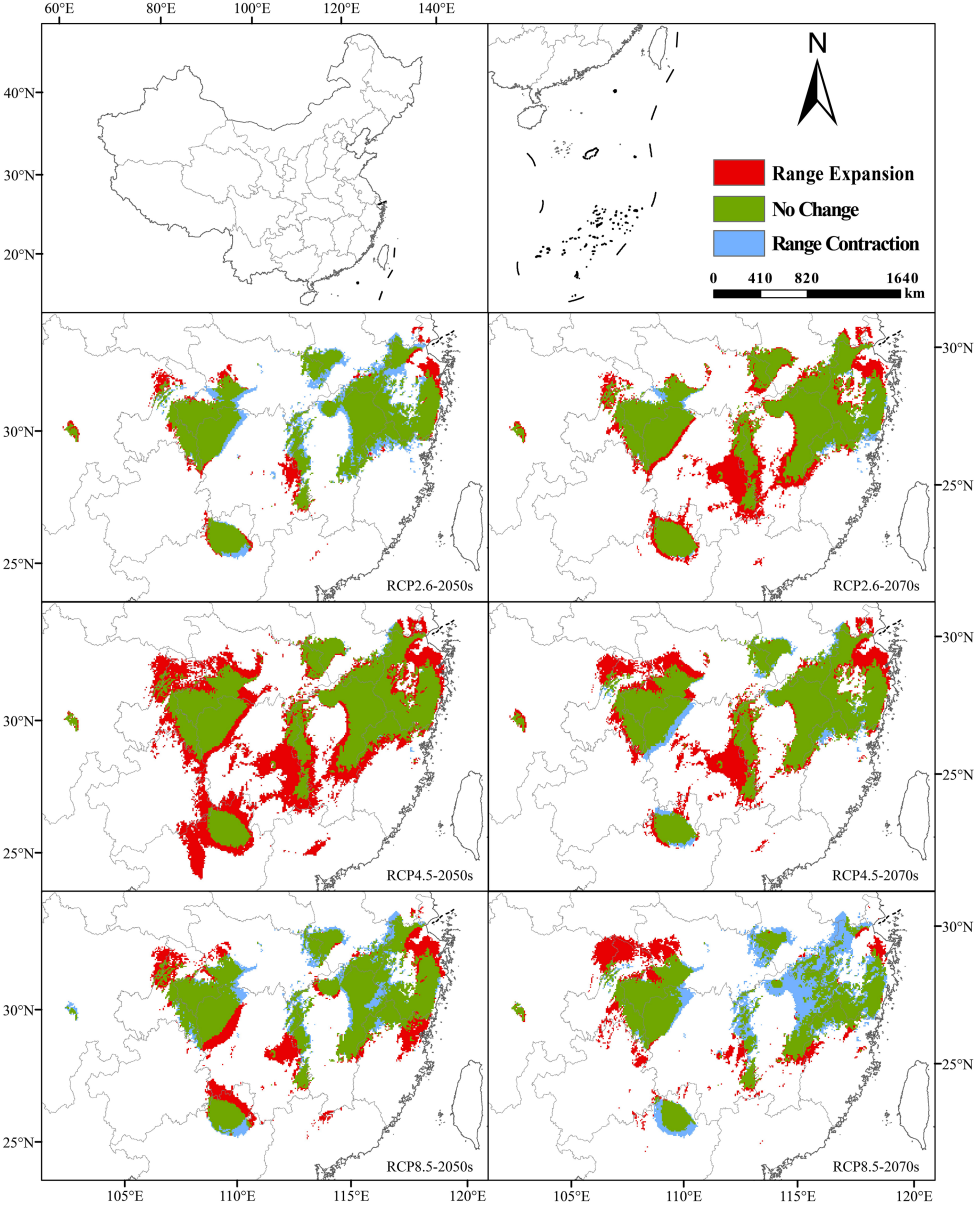
Spatial and geographical pattern of change in the highly suitable habitat for *Cirsium lineare* in the 2050s and 2070s compared with the current (red: expansion area, green: stable area, and blue: contraction area).

In addition, we also studied how the high‐suitability area shifted when the threshold is set to 0.60 (Figure 6b). Now the current centroid is located in Xiushui County, Jiangxi Province (114.9070°E, 28.8873°N). The range in variation of both RCP4.5 and RCP8.5 centroids is large, and their migration distance increases with the rising greenhouse gas concentration. In this century, under the three scenarios of RCP2.6, RCP4.5, and RCP8.5, the centroid was expected to migrate 31.45, 98.21, and 29.21 km, respectively, from the present to the 2050s, and the centroid RCP4.5–2050s is located in Liuyang City, Hunan Province (114.0010°E, 28.5006°N). The centroids RCP2.6–2050s and RCP8.5–2050s are located in Xiushui County, Jiangxi Province (114.5840°E, 28.8769°N), and Tonggu County, Jiangxi Province (114.6610°E, 28.7369°N). From the 2050s to the 2070s, the centroid, respectively, moves 25.81, 67.34, and 121.00 km under those three scenarios. That of RCP8.5–2070s is located in Yueyang City, Hunan Province (113.4580°E, 29.0125°N). Similarly, the total suitable area for *C. lineare* will also expand westward and northward (Figure 9).

## 4 DISCUSSION

The MaxEnt model is a commonly used model to study the spatial distribution of species. The use of species distribution data and environmental data imported into MaxEnt to analyze species distribution patterns over time and under different scenarios has a wide range of applications in fields such as ecology. In this study, based on the information of *C*. *lineare* samples, MaxEnt model was used to predict this plant's suitable area in China. The optimized model's ΔAICc = 0 and AUC > 0.8 indicate it has high accuracy and a robust fitting effect, supporting the reliability of our experimental results.

Being a perennial herb, *C*. *lineare*'s growth and biomass accumulation are affected by its local environment (Marshall et al., 2018). Here, we used the MaxEnt model to predict the distribution of *C. lineare* in different periods and under different scenarios and also studied the impact of environmental variables on this plant's distribution.

According to the model operation output, we analyzed the ordering of jackknife test results and contribution rates. Among the environmental variables that influenced the distribution of *C. lineare*, bio6 has the greatest impact on this plant's growth, with a contribution rate of 76.8%. The top three leading environmental factors are bio6, bio12, and bio2; hence, evidently, the distribution of *C. lineare* is primarily determined by the minimum temperature of the coldest month, annual precipitation, and the mean diurnal temperature range. At the same time, modern research has shown that changes in the spatial distribution of plants due to climate change‐induced environmental changes are mostly related to temperature and precipitation during their growth stages (Root et al., 2003; Tang et al., 2021; Zhang et al., 2021). Crucially, the experimental results show that the suitable distribution areas for *C. lineare* will gradually expand to the west and north in the future, and its total suitable distribution area is mainly distributed in East China, Central China, southeast of Northwest China, east of Southwest China, and South China in the 2070s. The total suitable area is projected to increase from 22,209,400 km^2^ at present to 24,672,100 km^2^ under the RCP8.5 scenario in the 2070s. Like most plants, with higher greenhouse gas emission concentrations, the total suitable area of *C. lineare* increases (i.e., positively correlated). With rising greenhouse gas emissions worldwide, the warming effect will gradually intensify, and China's climate is expected to also transition to a warm and humid climate (Yan et al., 2021). When that is reached, the environment should be very suitable for the growth of *C. lineare* and its populations. In addition, due to variation in the concentration of greenhouse gas emissions, the spatial pattern and centroid trajectory of the total and highly suitable areas for *C. lineare* will be inconsistent. In the spatial pattern maps (Figures 8 and 9), red indicates the expansion of habitat, green indicates it is unchanged, and blue indicates shrinking habitat areas.

With the continuous changes over time and among climate scenarios, the migration of the centroid position will also exhibit a certain pattern of change. The centroid of the total suitable area for *C. lineare* is currently located in Dingcheng District, Changde City, Hunan Province (111.8140°E, 29.0583°N), while that of highly suitable area is located in Xiushui County, Jiangxi Province (114.9070°E, 28.8873°N). The centroids in differing periods and scenarios are located at the north or west side of the current centroid; that is, the centroid moves toward high altitude or high latitude. From the prediction results, under the influence of future climate conditions, the spatial location change of the total suitable area is nearly consistent with the shift of the centroid: expanding to the west and north in tandem. This finding is consistent with other work showing that, in response to continual global warming, most species will gradually migrate to new habitats at high elevation or latitude (or both), in trying to adapt to their novel natural environment (Abdelaal et al., 2019; Fourcade et al., 2014; Lamprecht et al., 2018). Under future climate change, 20% of species covering the global area will be at risk of extinction, and about 15%–37% of species will be endangered (Lambers, 2015). At the same time when ecosystems at lower elevations are destroyed, higher elevations can still maintain good ecosystems, and species habitats will migrate to areas that are more suitable for survival. Likewise, the predicted suitable habitat range of many species, including medicinal plants, will also change with climate change (Casseau et al., 2015; Guo et al., 2016; You et al., 2018; Zhao et al., 2018). In general, when establishing protected areas and artificial cultivation bases for species, in addition to considering the current suitable areas, it is also necessary to formulate reasonable conservation and management programs and plan the best planting areas according to the effects of future climate change and dominant environmental factors.

However, first, we used the MaxEnt model to analyze the relationship between the distribution of potentially suitable habitat for *C. lineare* and climate data, and the results are only theoretical speculation. Second, the nine environmental variables we selected cannot completely replace the joint impact arising from all influencing factors. Given that most *Cirsium* Mill. plant species have crown hairs, wind speeds will also affect their distribution range (Qin et al., 2017). Third, plants grow in a complex ecosystem where species are distributed the result of their interaction with nature and long‐term adaptation (Kendal et al., 2012). In addition to temperature and precipitation, solar radiation, soil (Mao et al., 2022), and human activities (Lamprecht et al., 2018; Zangiabadi et al., 2021) are capable of substantially impacting the distribution of these species. Therefore, the proper combination of multiple environmental variables applied to habitat suitability simulation exercises can improve the overall accuracy standard of the model, to render the modeling results more accurate and realistic.

## 5 CONCLUSION

In this study, the MaxEnt model and climate data of different periods were used to theoretically predict the suitability distribution of *Cirsium lineare* in China under future climate change. Among the 19 environmental variables, we selected nine weakly correlated variables, such as average annual temperature and rainfall for this modeling research, among which bio2, bio6, and bio12 have the greatest impact on the growth of *C. lineare*. Over time, compared with the current distribution range of *C. lineare*, its distribution range will expand under future climate change. From the perspective of greenhouse gas emission concentrations, the higher they are, the larger the total suitable area will be. In general, the total suitable distribution area of *C. lineare* in China will gradually enlarge toward the west and north. In view of ongoing and anticipated climate change trends, our experimental results can play a valuable role in formulating an effective climate change adaptation and protection strategy for *C. lineare* wild populations.

## AUTHOR CONTRIBUTIONS

**Hu‐Qiang Fang:** Formal analysis (equal); methodology (equal); writing – original draft (equal). **Peng‐Fei Zhang:** Validation (equal). **Shao‐Wei Xu:** Validation (equal). **Teng Xu:** Visualization (equal). **Bing He:** Formal analysis (equal). **En Wang:** Validation (equal). **Chang‐Wu Dong:** Supervision (equal). **Qing‐Shan Yang:** Conceptualization (equal); supervision (equal).

## CONFLICT OF INTEREST STATEMENT

The authors declare no conflict of interest.

## Data Availability

Geographical data and maps are available via ZENODO http://doi.org/10.5281/zenodo.7978352.
